# Expression of voltage-gated Ca^2+^ channels, Insp_3_Rs, and RyRs in the immature mouse ovary

**DOI:** 10.1186/s13048-022-01015-y

**Published:** 2022-07-22

**Authors:** Daniel Bahena-Alvarez, Diana Millan-Aldaco, Ruth Rincón-Heredia, Nancy Escamilla-Avila, Arturo Hernandez-Cruz

**Affiliations:** 1 Departamento Neurociencia Cognitiva, Instituto de Fisiología Celular, Universidad Nacional Autónoma de México, Circuito de la Investigación Científica. Col. UNAM, Ciudad Universitaria, CP 04510 México CDMX, México; 2 Unidad de Imagenología, Instituto de Fisiología Celular, Universidad Nacional Autónoma de México, Ciudad Universitaria, Avenida Universidad 3000, México City, CDMX, 04510 México; 3 Laboratorio Nacional de Canalopatías, Instituto de Fisiología Celular, Universidad Nacional Autónoma de México, Ciudad Universitaria, Avenida Universidad 3000, México City, CDMX, 04510 México

**Keywords:** Follicle, Granulosa cells, Oocyte, Calcium channels, Calcium signaling, Ovarian maturation. Immunofluorescence

## Abstract

**Background:**

The postnatal mammalian ovary undergoes a series of changes to ensure the maturation of sufficient follicles to support ovulation and fecundation over the reproductive life. It is well known that intracellular [Ca^2+^]_i_ signals are necessary for ovulation, fertilization, and egg activation. However, we lack detailed knowledge of the molecular identity, cellular distribution, and functional role of Ca^2+^ channels expressed during folliculogenesis. In the neonatal period, ovarian maturation is controlled by protein growth factors released from the oocyte and granulosa cells. Conversely, during the early infantile period, maturation becomes gonadotropin-dependent and is controlled by granulosa and theca cells. The significance of intracellular Ca^2+^ signaling in folliculogenesis is supported by the observation that mice lacking the expression of Ca^2+^/calmodulin-dependent kinase IV in granulosa cells suffer abnormal follicular development and impaired fertility.

**Results:**

Using immunofluorescence in frozen ovarian sections and confocal microscopy, we assessed the expression of high-voltage activated Ca^2+^ channel alpha subunits and InsP_3_ and ryanodine receptors in the postnatal period from 3 to 16 days. During the neonatal stage, oocytes from primordial and primary follicles show high expression of various Ca^2+^-selective channels, with granulosa and stroma cells expressing significantly less. These channels are likely involved in supporting Ca^2+^-dependent secretion of peptide growth factors. In contrast, during the early and late infantile periods, Ca^2+^ channel expression in the oocyte diminishes, increasing significantly in the granulosa and particularly in immature theca cells surrounding secondary follicles.

**Conclusions:**

The developmental switch of Ca^2+^ channel expression from the oocytes to the perifollicular cells likely reflects the vanishing role of the oocytes once granulosa and theca cells take control of folliculogenesis in response to gonadotropins acting on their receptors.

**Supplementary Information:**

The online version contains supplementary material available at 10.1186/s13048-022-01015-y.

## Background

Ion channels perform a diversity of essential functions in mammalian cells. However, we still lack a detailed description of most ovarian ion channels' molecular identity, cellular distribution, and functional role [3]. This information is required to design specific treatments that modulate ion channel function to control fertility and prevent or ameliorate ovarian diseases.

The resting state of most cells is characterized by a low cytoplasmic Ca^2+^ concentration ([Ca^2+^]_i_) (∼100 nM). [Ca^2+^]_I_ transient elevations, termed Ca^2+^ signals, carry out many specific functions [2]. The onset of a Ca^2+^ signal results from Ca^2+^ moving into the cytoplasm, either by transmembrane Ca^2+^ influx or Ca^2+^ release from intracellular stores. Voltage-gated Ca_V_ channels can mediate Ca^2+^ influx [38]. The high-voltage activated (HVA) group comprises four L-type channel subtypes (Ca_V_1.1 to 1.4) and P/Q, N, and R-type channel subtypes (Ca_V_2.1 to 2.3). The low-voltage activated (LVA) group includes three T-type channel subtypes (Ca_V_3.1 to 3.3). Intracellular Ca^2+^-release channels, Inositol triphosphate (InsP_3_R), and Ryanodine receptors (RyR) are ligand-gated Ca^2+^-permeable channels from the smooth endoplasmic reticulum (SER). Their role is to release into the cytoplasm some of the Ca^2+^ stored in the SER by the SERCA (smooth endoplasmic reticulum Ca^2+^-ATPase). Since Ca^2+^ opens both InsP_3_Rs and RyR, released Ca^2+^ initially participates in signal amplification (termed Ca^2+^-induced Ca^2+^-release). Ca^2+^ also acts as an antagonist at higher concentrations and helps to terminate Ca^2+^ signals [33]. Other ion channels relevant to the ovary are store-operated Ca^2+^ entry (SOCE), a Ca^2+^ influx mechanism activated when SER Ca^2+^ stores are emptied [3]. Here, the SER Ca^2+^ sensors are STIM1 and STIM2, and the plasma membrane (PM) Ca^2+^-selective channels are Orai1, 2, and 3 [27]. Another type of PM Ca^2+^-permeable channel is the TRPs, which by acting as a signal transducer, modulates both membrane potential and [Ca^2+^]_i_. Most TRP channels are Ca^2+^-permeable with varying Ca^2+^ selectivity [31].

In general, PM ion channels regulate membrane potential. Nonetheless, Ca^2+^ channels are unique because their activation also generates Ca^2+^ fluxes that change [Ca^2+^]_i_, a signal that controls migration, proliferation, growth, maturation, and hormone secretion. [Ca^2+^]_i_ is also intimately linked to apoptosis and cytotoxic cell death. In the ovary, [Ca^2+^]_i._ works as a universal activator of fertilization, egg activation, and egg-to-embryo transition [3]. However, very little is known about the role of Ca^2+^ channels (or any other channel) in folliculogenesis. A recent review mentions that the lack of connexin 43 (Cx43), a channel that mediates interactions between granulosa cells in mutant mice, impairs postnatal folliculogenesis and granulosa cell layer formation [3].

We recently described the varying expression of PM voltage-gated Ca^2+^ channels in the mouse ovary throughout the estrous cycle [1]. Here, we extended this study to the neonatal stage (PND 0-7), when the pituitary secretion of LH/FSH is deficient, and recruitment of primordial follicles, oocyte maturation, and early folliculogenesis depend exclusively on intra-ovarian autocrine and endocrine signals secreted by the oocyte and the granulosa cells [32, 34]. We also examined the early (PND 8-14) and late infantile (PND 15-20) stages: During the early stage, gonadotropin secretion begins when LH and FSH receptors commence appearing in granulosa and theca cells. After that, ovarian maturation and folliculogenesis become increasingly dependent on gonadotropins.

The role of intracellular Ca^2+^ signaling in folliculogenesis is supported by the observation that FSH increases [Ca^2+^]_i._ in granulosa cells [11, 35] and that a mutant mouse lacking Ca^2+^/calmodulin-dependent kinase IV (CAMK IV) in their granulosa cells suffer impaired fertility and abnormal follicular and luteal development [37]. It is vital to understand the role of Ca^2+^ selective channels in ovarian maturation since they likely define the continuum of follicle transitions, from primordial to preovulatory, and eventually to become competent for ovulation. It has been suggested that Ca^2+^ selective plasma membrane channels and intracellular Ca^2+^ release channels also have significant roles in ovulation and fertilization [3].

Here we compared the distribution and expression of voltage-gated Ca^2+^ channels and intracellular Ca^2+^ release channels InsP_3_ receptors (InsP_3_R), and ryanodine receptors (RyRs) in the ovary during the crucial transition from neonatal to early and late infantile periods [16, 33]. Hopefully, information on the differential expression of ovarian ion channels will translate into a better understanding of ion channels' role in the recruitment of primordial follicles and their gradual transition to primary, secondary, and early antral follicles.

## Results

### Expression of Ca^2+^-selective ion channels in the neonatal stage (PND 3)

#### Ca_V_1.2 *(α1C)* immunostaining

**Fig. 1:**
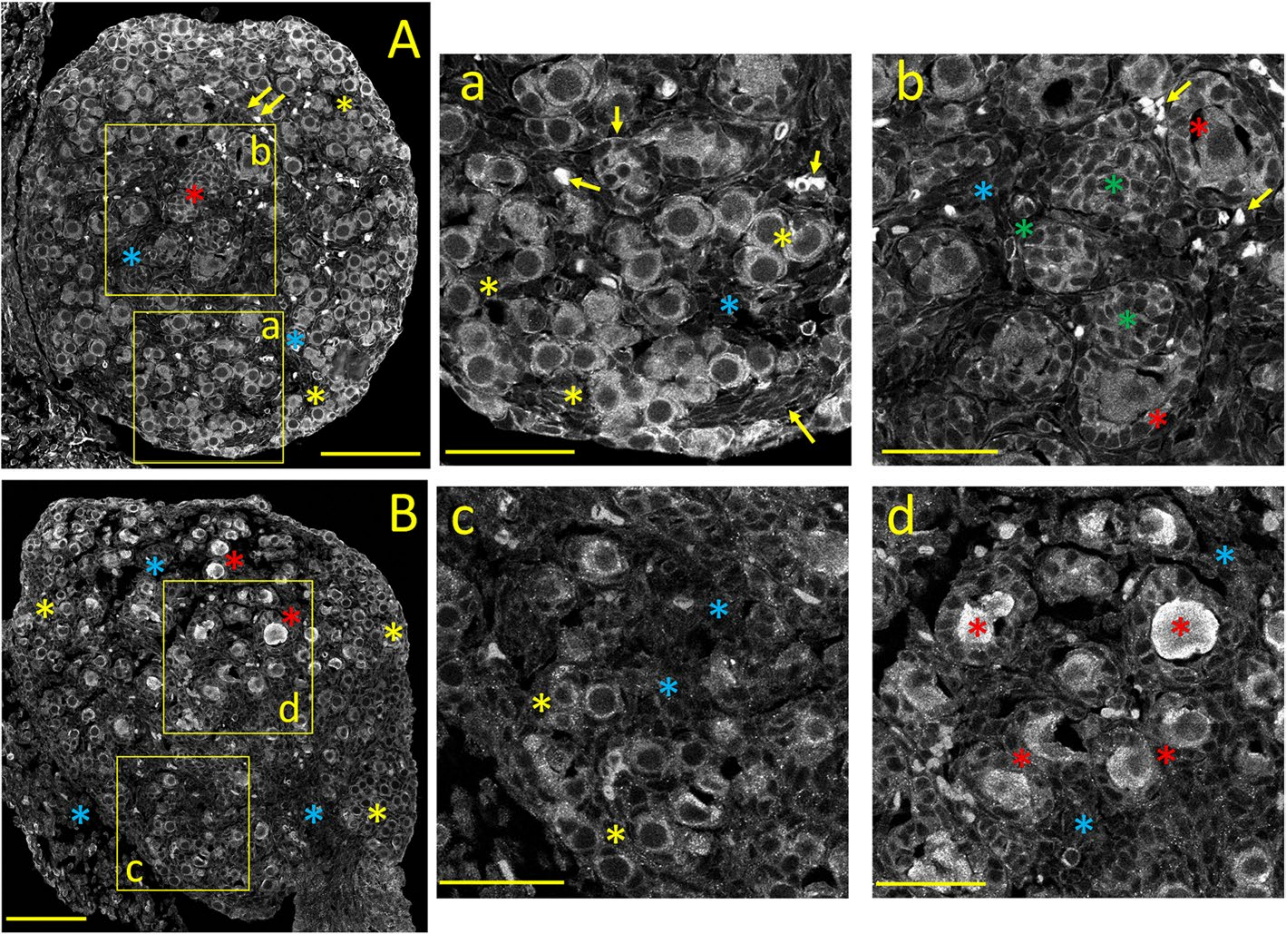
Tissue distribution of specific immunolabelling for Ca_V_1.2 and Ca_V_1.3 voltage-gated Ca^2+^ channels in the neonatal ovary (PND 3). **A:** Ca_V_1.2 specific immunostaining. **a, b:** Enlarged images from the square areas indicated in **A.** *yellow and red asterisks:* strong cytoplasmic staining of oocytes from primordial and primary follicles, respectively. *green asterisks:* granulosa cells, stained at the plasma membrane. *yellow arrows:* strongly Ca_V_1.2-positive cells, possibly autonomic neurons, and non-myelinated nerve fibers. *blue asterisks:* unstained stromal cells. **B:** Ca_V_1.3 specific immunostaining. **c, d:** Enlarged images from the square areas indicated in **B.** *yellow and red asterisks:* stronger cytoplasmic staining of primordial versus primary follicles, respectively. *blue asterisks:* unstained stromal cells. Calibration bar: 100 μm (A, B); 50 μm (a, b, c, d)

The expression pattern of Ca_V_1.2 Ca^2+^ channels subunits, which produce HVA L-type, sustained, dihydropyridine-sensitive Ca^2+^ currents, is shown in Fig. 1A. The neonatal ovary is characterized by a lack of boundary between the cortex and medulla. Numerous primordial follicles, surrounded by a single layer of flattened (squamous) pre-granulosa cells, are clustered near the periphery (Fig. 1A and a: *yellow asterisks*). Primary follicles, characterized by an oocyte surrounded by a single layer of immature granulosa cells, are seen near the medulla's central core (Fig. 1A and b: *red asterisks*). Primordial and primary follicular oocytes, and to a lesser extent, granulosa cells, display robust cytoplasmic Ca_V_1.2 staining, while stromal cells between follicles are weakly or not stained *(blue asterisks;* see also Fig. 10A)*.* Bundles of thin, Ca_V_1.2-positive processes resembling non-myelinated nerve fibers are seen close to small intensely Ca_V_1.2-positive cells, possibly autonomic neurons (Fig. 1A and a, b*: yellow arrows).* Ca_V_1.2-positive nerve fiber-like processes were described in the mature ovary [1].

#### Ca_V_1.3 *(α1D)* immunostaining

The neonatal ovarian expression of Ca_V_1.3 Ca^2+^ channel alpha subunits, which also generate HVA, L-type Ca^2+^ currents, is illustrated in Fig. 1B. In contrast to the relatively uniform staining of Ca_V_1.2 (Fig. 1A), the most salient feature of Ca_V_1.3 immunostaining is the more robust cytoplasmic labeling of primary follicles (Fig. 1B, d, *red asterisks*) compared to the primordial ones (Fig. 1B*,* c, *yellow asterisks*; see also Fig. 10B). The staining intensity varies considerably among oocytes (Fig. 1d, *red asterisks).* Large areas of weakly stained stromal cells fill the spaces between follicles (Fig. 1B*,* and c, d: *blue asterisks*)*.*

#### Ca_V_2.1 *(α1A*) immunostaining

**Fig. 2:**
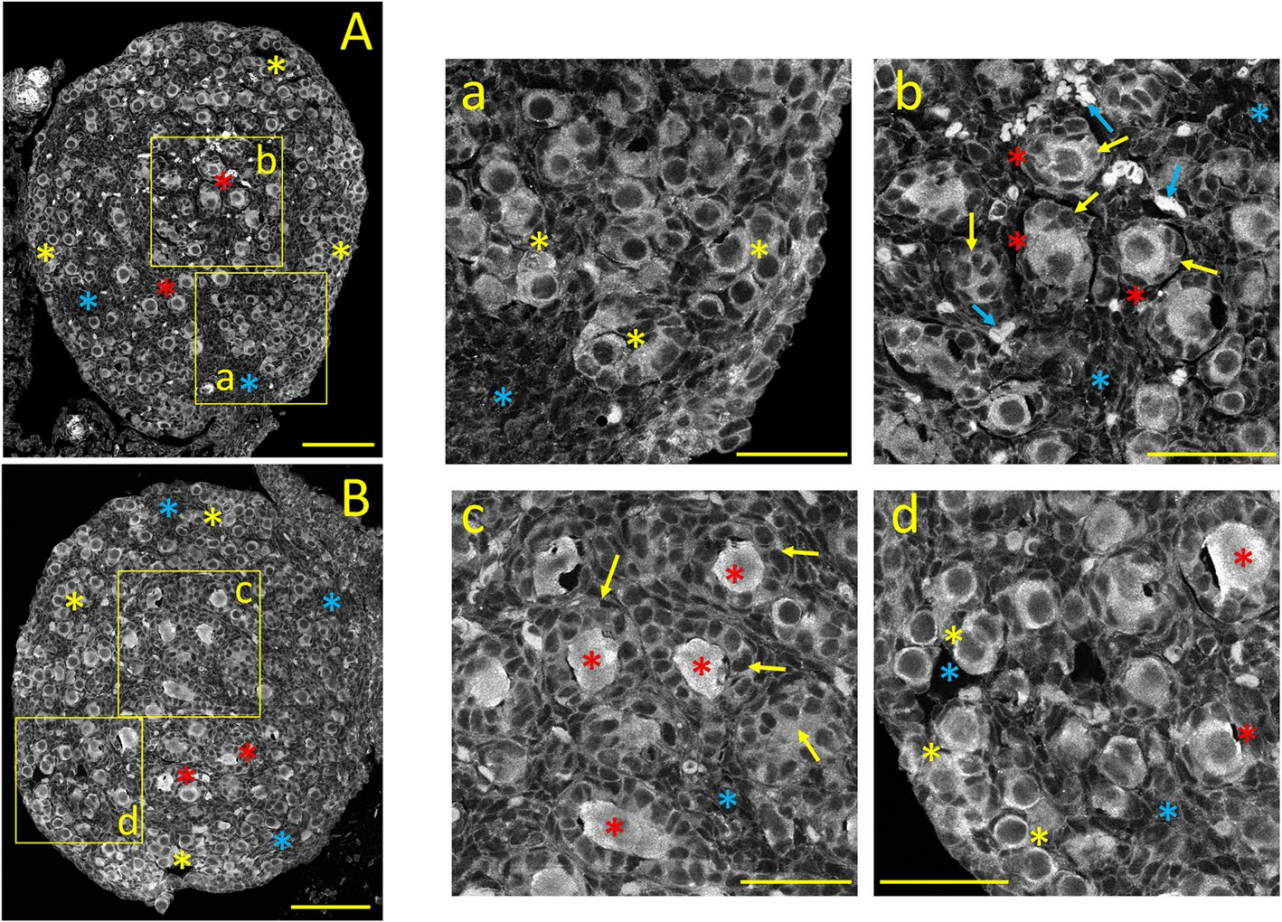
Tissue distribution of specific immunolabelling for Ca_V_2.1 and Ca_V_2.2 voltage-gated Ca^2+^ channels in the neonatal ovary (PND 3). **A:** Ca_V_2.1 specific immunostaining. **a**,** b:** Enlarged images from the square areas indicated in **A**. *yellow and red asterisks:* Strong staining of primordial and primary follicles, respectively. *yellow arrows:* Ca_V_2.1 positive granulosa cells surrounding primary follicles. *blue arrows:* Intensively Ca_V_2.1 immunoreactive cells (possibly autonomic neurons). *blue asterisks:* unstained stromal cells. **B:** Ca_V_2.2 specific immunostaining. **c**,** d:** Enlarged images from the square areas indicated in **B**. *yellow* and *red asterisks:* Strong Ca_V_2.2 immunostaining of primordial and primary follicles, respectively. *yellow arrows:* positive granulosa cells surrounding primary follicles *blue asterisks:* unstained stromal cells. Calibration bar: 100 μm (A, B); 50 μm (a, b, c, d)

Ca_V_2.1 Ca^2+^ channel subunits generate HVA, P/Q-type sustained Ca^2+^ currents, primarily responsible for fast synaptic transmission in the nervous system [21]. The unexpected expression of this neuronal Ca^2+^-channel subunit was first reported in the *corpora lutea* and stromal cells from the mature ovary [1]. The Ca_V_2.1 immunostaining pattern in the neonatal ovary is shown in Fig. 2A. Ca_V_2.1 expression is similar to that of Ca_V_1.3, including the stronger cytoplasmic labeling of oocytes from primary and primordial follicles (Fig. 2A and a, b: *yellow* and *red asterisks,* respectively; see also Fig. 10C). Cuboidal granulosa cells surrounding primary follicles display milder cytoplasmic staining (Fig. 2b: *yellow arrows*). Strongly Ca_V_2.1 positive (possible autonomic nerve cells) are seen near the medulla's central core (Fig. 2b: *blue arrows)*. Large interfollicular areas are filled with unstained stromal cells (Fig. 2A, a, b; *blue asterisks*).

#### Ca_*V*_2.2 *(α1B)* immunostaining

Ca_V_2.2 Ca^2+^-channel subunits, which generate HVA, N-type sustained Ca^2+^ currents, are localized primarily on nerve terminals, dendrites, and neuroendocrine cells [5]. Nonetheless, we previously demonstrated that these Ca^2+^-channel subunits are present in perifollicular smooth muscle cells from the mature ovary [1]. The expression pattern of Ca_V_2.2 in the neonatal ovary is shown in Fig. 2B. Similar to Ca_V_2.1, oocytes from primary and primordial follicles are strongly Ca_V_2.2 immunostained (Fig. 2B and c, d; *yellow* and *red asterisks,* respectively; see also Fig. 10D). The cuboid granulosa cells surrounding primary follicles oocytes show moderate cytoplasmic staining (Fig. 2c: *yellow arrows*). Weakly stained stromal cells fill the areas free of follicles (Fig. 2B, c, d: *blue asterisks*).

#### InsP_3_Rs immunostaining

**Fig. 3:**
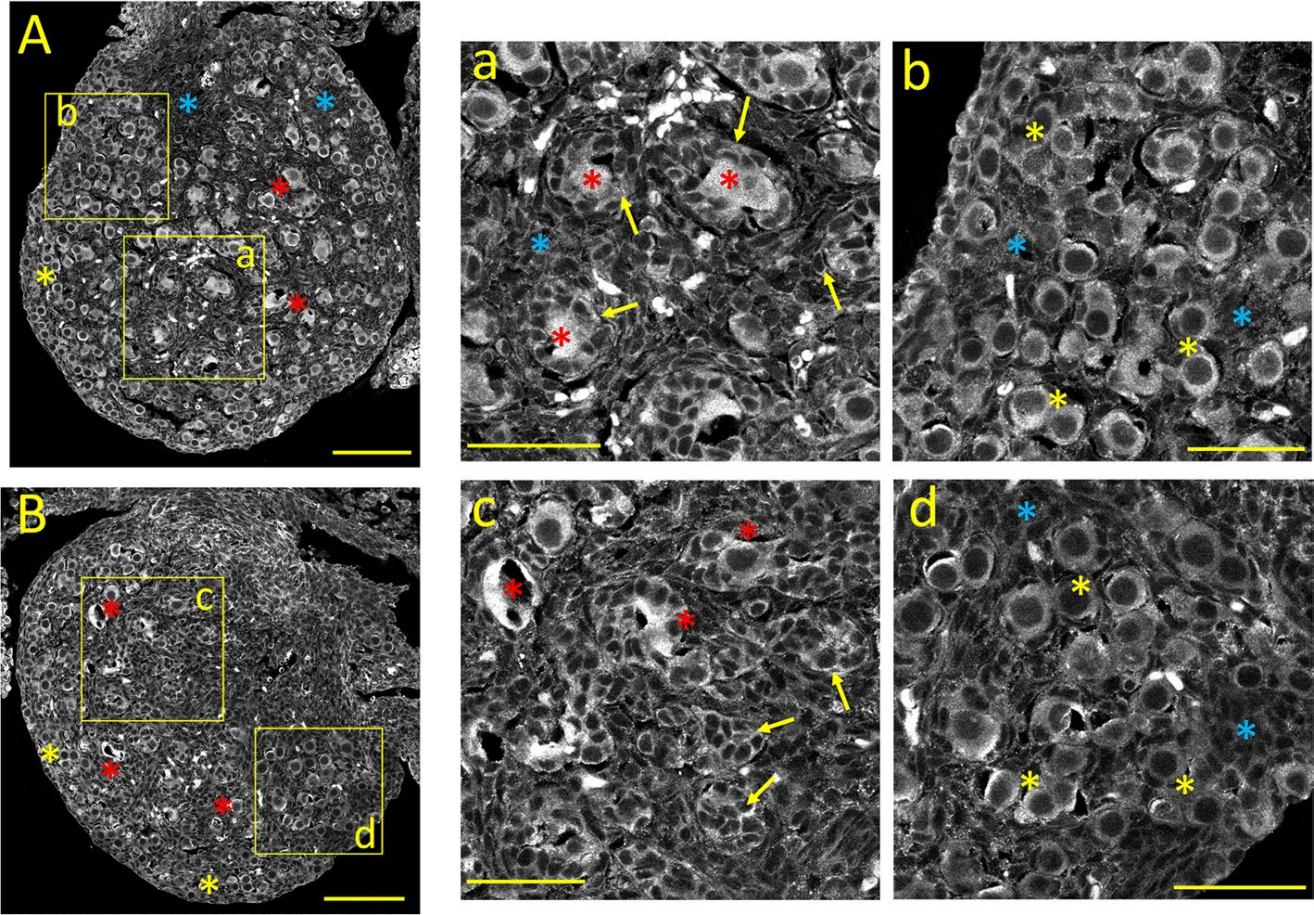
Tissue distribution of specific immunolabelling for Ca^2+^ release channels InsP_3_Rs and RyR in the neonatal ovary (PND 3). **A:** InsP_3_R-specific immunostaining. **a**,** b:** Enlarged images from the square areas indicated in **A**. *yellow* and *red asterisks:* strongly InsP_3_R-positive oocytes from primordial and primary follicles, respectively. *yellow arrows:* granulosa cells surrounding primary follicles display labeling at the plasma membrane. *blue asterisks:* unstained stromal cells. **B:** RyR-specific immunostaining. **c**,** d:** Enlarged images from the square areas indicated in **B**. *yellow asterisks:* primordial follicles show moderate cytoplasmic RyR staining. *red asterisks:* primary follicles. *yellow arrows:* granulosa cells from primary follicles display moderate labeling near the plasma membrane. *blue asterisks:* unstained stromal cells. Calibration bar: 100 μm (A, B), 50 μm (a, b, c, d)

The expression pattern of the Ca^2+^ release channel InsP_3_Rs in the neonatal ovary is shown in Fig. 3A. Overall, InsP_3_R labeling resembles that of Ca_V_1.2 Ca^2+^-channel subunits. Oocytes from primordial and primary follicles show distinct cytoplasmic staining, with primordial oocytes showing higher immunoreactivity (Fig. 3A and a, b; *yellow* and *red asterisks,* respectively; see also Fig. 10E). Granulosa cells from primary follicles display moderate labeling, especially close to the plasma membrane (Fig. 3a, *yellow arrows*). Stromal cells between follicles almost lack InsP_3_R staining (*blue asterisks*).

#### RyRs immunostaining

The distribution of RyRs in the neonatal ovary is shown in Fig. 3B. In general, the expression pattern of these Ca^2+^ release channels is similar to that of Ca_V_1.3, Ca_V_2.1, and Ca_V_2.2, Oocytes from primordial follicles display mild RyR cytoplasmic staining (Fig. 3B and d, *yellow asterisks*). In contrast, oocytes from primary follicles show stronger RyR staining. However, this varies considerably among oocytes: some primary oocytes are weakly stained, while others show uneven, intense cytoplasmic staining (Fig. 3B, and c: *red asterisks*). Granulosa cells from primary follicles show distinct cytoplasmic RyR immunostaining (Fig. 3c, *yellow arrows*), while stromal cells are stained weakly or not stained (*blue asterisks*, Fig. 3d; see also Fig. 10F). The mean immunofluorescence intensities of the different ovarian structures at postnatal day three are summarized in Fig. 10A-F.

### Expression of Ca^2+^-selective ion channels during the early infantile period (PND 8)

#### Ca_V_1.2 *(α1C)* immunostaining

**Fig. 4:**
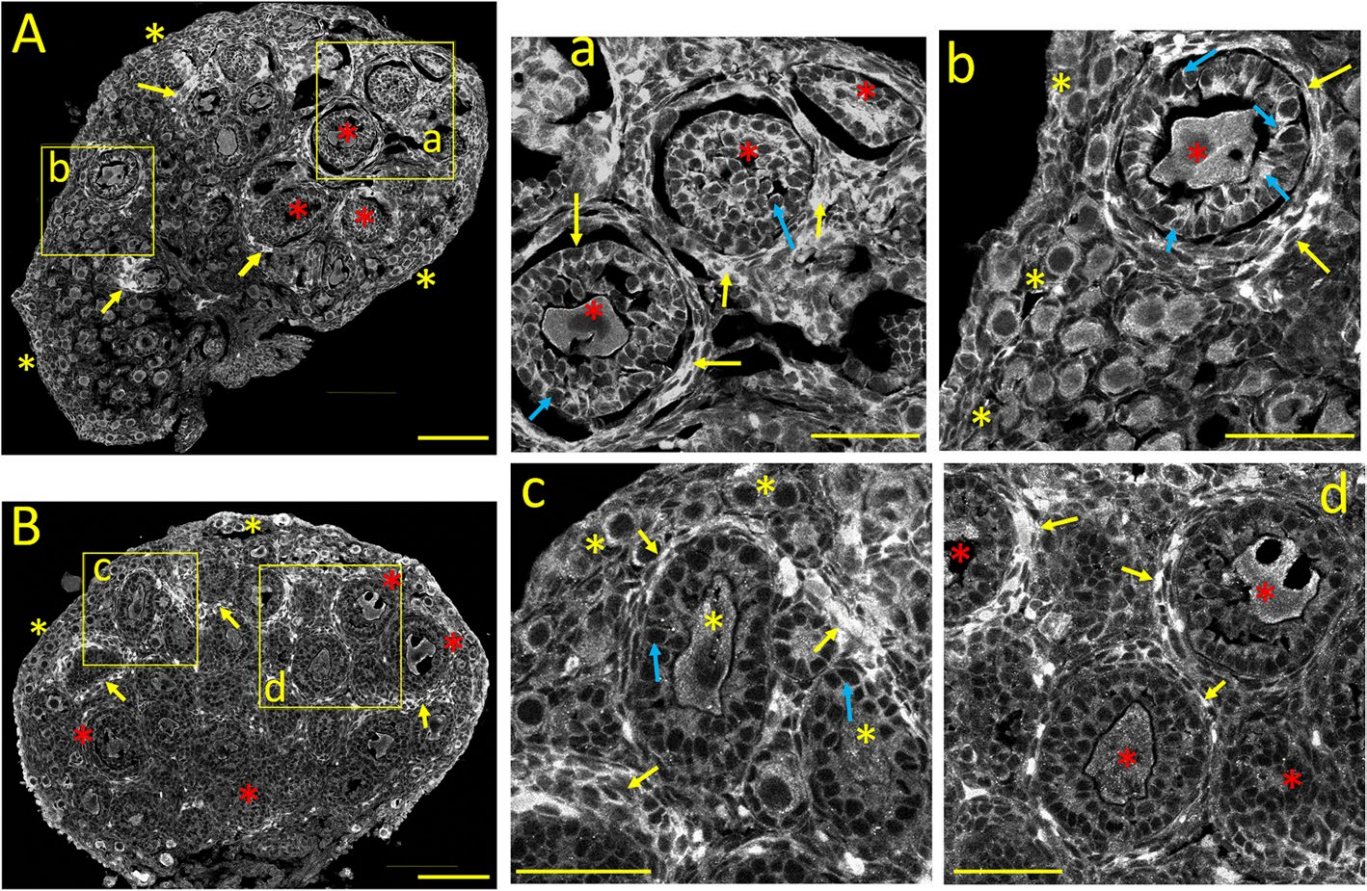
Tissue distribution of specific immunolabelling for Ca_V_1.2 and Ca_V_1.3 voltage-gated Ca^2+^ channels during the early infantile ovary (PND 8). **A:** Ca_V_1.2 specific immunostaining. **a**,** b:** Enlarged images from the square areas indicated in **A**. yellow and red asterisks: remaining oocytes from primordial, primary, and secondary, early antral follicles, respectively, are moderately stained. *blue arrows:* granulosa cells from these follicles show cytoplasmic staining at the plasma membrane and close to the oocyte. *yellow arrows:* patches of perifollicular cells, possibly immature theca cells are strongly Ca_V_1.2-positive. **B:** Ca_V_1.3 specific immunostaining. **c**,** d:** Enlarged images from the square areas indicated in **B**. *yellow and red asterisks:* moderate Ca_V_1.3 immunolabeling of oocytes and granulosa cells from primordial and primary follicles and secondary and early antral follicles, respectively. Ca_V_1.3 positive oocytes and granulosa cells from secondary and early antral follicles. *blue arrows:* Ca_V_1.3 immunoreactivity of granulosa cells near the plasma membrane. *yellow arrows:* strongly Ca_V_1.3 positive perifollicular cells surrounding primary, secondary and early antral follicles*.* Calibration bar: 100 μm (A, B); 50 μm (a, b, c, d)

During the early infantile stage (PND 8-14), the residual primordial follicles are pushed towards the periphery (Fig. 4A and b, *yellow asterisks*) by the formation and growth of primary, secondary, and early antral follicles underneath (Fig. 4A and a, b; *red asterisks*). At this stage, oocytes of primordial and primary follicles show moderate immunostaining compared to the neonatal stage. Granulosa cells from primary and secondary follicles show distinct cytoplasmic Ca_V_1.2 labeling, particularly in the plasma membrane and the pole closer to the oocyte (4a, b; *blue arrows*). Interestingly, groups of perifollicular cells (likely immature theca cells) begin to display distinct immunostaining at this stage (4A and 4a, b; *yellow arrows,* see also Fig. 10A_1_).

#### Ca_V_1.3 *(α1D)* immunostaining

The ovarian expression pattern of Ca_V_1.3 at the early infantile stage is shown in Fig. 4B. Here, the oocytes and granulosa cells from primordial and primary follicles display moderate Ca_V_1.3 immunolabeling (Fig. 4B and c, d: *yellow asterisks*), while oocytes and granulosa cells from secondary and early antral follicles express milder staining (Fig. 4B and d: *red asterisks*; see also Figure 10B_1_). Ca_V_1.3 immunoreactivity of granulosa cells is stronger near the plasma membrane (Fig. 4c: *blue arrows*). Conversely, clusters of flat perifollicular cells (likely immature theca cells) surrounding primary, secondary and early antral follicles show the most robust Ca_V_1.3 immunoreactivity (Fig. 4B and c, d: *yellow arrows*; Fig. 10B_1_).

#### Ca_V_2.1 *(α1A)* immunostaining

**Fig. 5:**
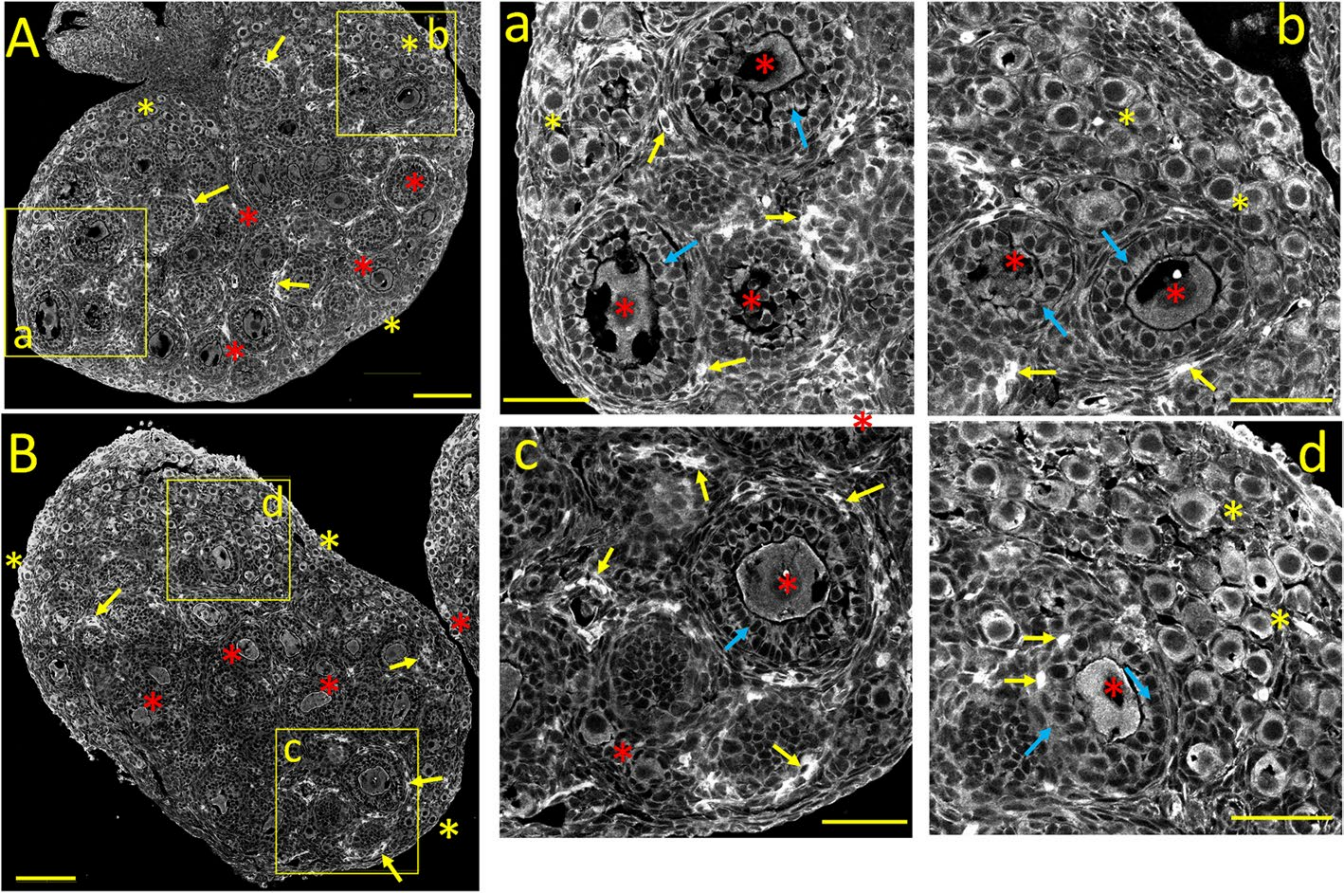
Tissue distribution of specific immunolabelling for Ca_V_2.1 and Ca_V_2.2 voltage-gated Ca^2+^ channels in the early infantile ovary (PND 8)***.*** **A:** Ca_V_2.1 specific immunostaining. **a**,** b:** Enlarged images from the square areas indicated in **A**. *yellow asterisks:* weak staining of few primordial oocytes near the plasma membrane. *red asterisks:* distinct Ca_V_2.1 staining of oocytes and granulosa cells from primary and secondary follicles. *blue arrows:* cytoplasmic staining of granulosa cells close to the plasma membrane in primary follicles. *yellow arrows:* flat, perifollicular stromal cells next to secondary and early antral follicles show the strongest Ca_V_2.1 immunolabeling. **B:** Ca_V_2.2 specific immunostaining. **c**,** d:** Enlarged images from the square areas indicated in **B**. Ca_V_2.2-specific staining is moderate throughout the ovary. *yellow asterisks: few* remaining primordial follicles*. red asterisks:* oocytes showing moderate Ca_V_2.2 immunostaining: *blue arrows:* weakly stained granulosa cells surrounding oocytes from primary follicles. *yellow arrows:* patches of perifollicular cells around secondary and early antral follicles show the strongest Ca_V_2.2 labeling. Calibration bar: 100 μm (A, B); 50 μm (a, b, c, d)

The expression pattern of Ca_V_2.1 Ca^2+^ channel subunits at the early infantile stage is exemplified in Fig. 5A. Moderately stained primordial oocytes are visible at the periphery (Fig. 5A, b, c*: yellow asterisks*). For the most part, the ovarian Ca_V_2.1 staining resembles that of Ca_V_1.3 at the same stage (see Fig. 10C_1_. Nonetheless, when PND3 and PND8 are compared, an outstanding feature of the latter is the distinctive Ca_V_2.1 staining of oocytes and granulosa cells from primary, secondary, and preantral follicles (Fig. 5A and a-b: *red asterisks*). In granulosa cells, Ca_V_2.1 staining is localized near the plasma membrane (*blue arrows*). Nonetheless, the strongest labeling is seen on flat theca and somewhat less in the stromal cells adjacent to secondary and early antral follicles (Fig. 5A and a-b: *yellow arrows*; see Fig. 10C_1_).

#### Ca_V_2.2 *(α1B)* immunostaining

The expression pattern of Ca_V_2.2 in the early infantile stage of the ovary is shown in Fig. 5B. Ca_V_2.2-specific staining throughout the ovary is moderate and relatively uniform, including oocytes from primordial, primary, secondary, and early antral follicles, stromal, granulosa, and most immature theca cells (Fig. 5B and c, d; see Fig. 10D_1_). This expression pattern differs from the neonatal stage, where strongly labeled oocytes are surrounded by a layer of faintly stained cuboid granulosa cells (Fig. 2B, c, d). Mildly stained granulosa cells are seen surrounding primary follicles (Fig. 5B and c, d: *blue arrows*). In contrast, patches of flat, elongated cells forming incomplete layers around secondary and early antral follicles show strong Ca_V_2.2-positivity (Fig. 5B and c, d: *yellow arrows*). These Ca_V_2.2-positive cells are likely precursors of the perifollicular cells, which co-stain with the smooth-muscle cell marker SMA*α* in mature ovarian follicles [1].

#### InsP_3_R immunostaining

**Fig. 6:**
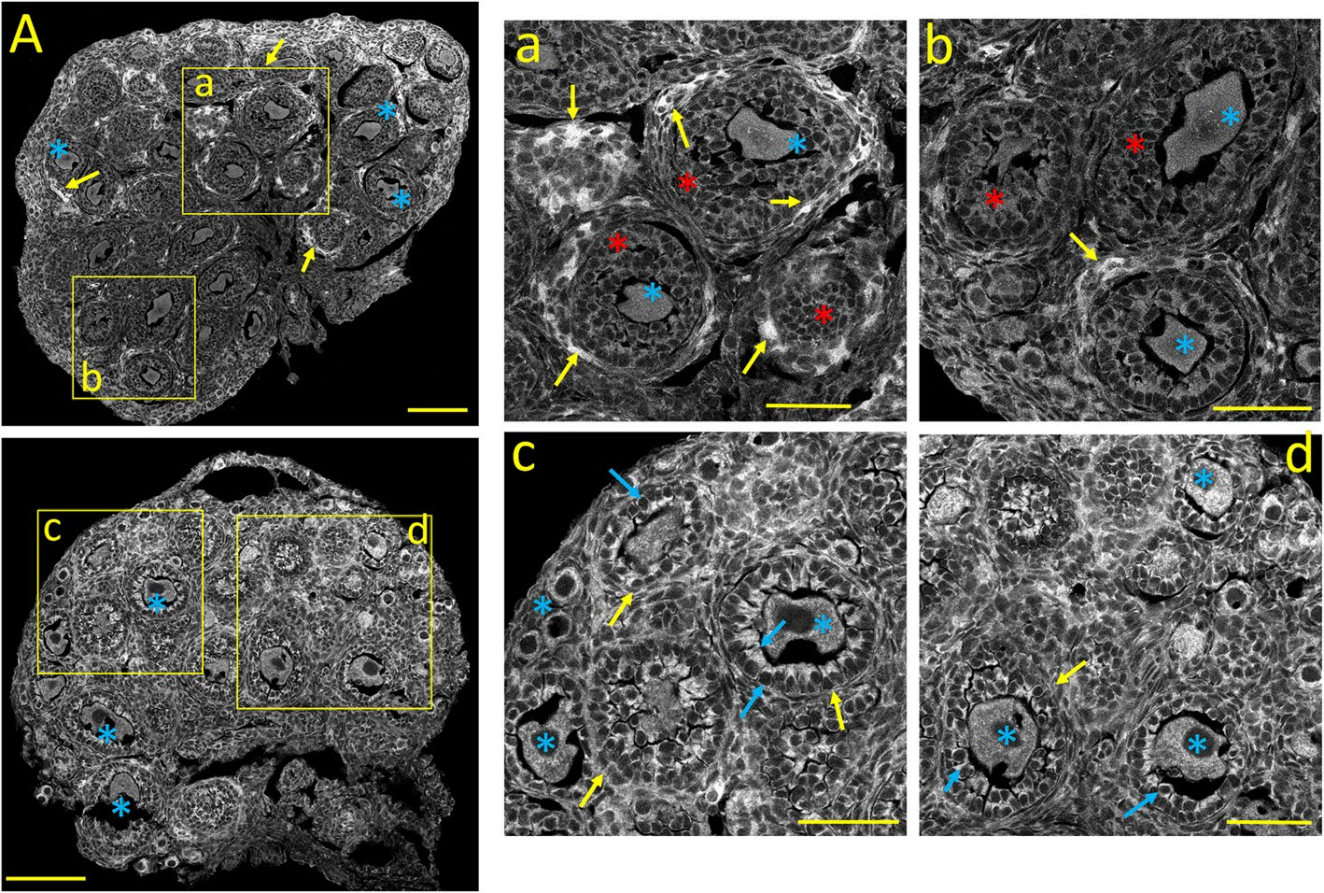
Tissue distribution of specific immunolabelling for Ca^2+^ release channels InsP_3_Rs and RyR in the early infantile ovary (PND 8). **A:** InsP_3_R-specific immunostaining. **a**,** b:** Enlarged images from the square areas indicated in **A**. *blue and red asterisks:* Oocytes and granulosa cells, respectively, from primary to early antral follicles, show moderate cytoplasmic labeling. *yellow arrows:* patches of perifollicular cells surrounding secondary and early antral follicles show strong immunostaining. **B:** RyR-specific immunostaining. **c**,** d:** Enlarged images from the square areas indicated in **B**. The whole ovary expresses RyRs. *blue asterisks:* oocytes, from primordial to early antral follicles, show punctate cytoplasmic staining. *blue arrows:* granulosa cells from the same follicles show distinct cytoplasm labeling at the plasma membrane closer to the oocyte. *yellow arrows:* perifollicular cells are not or weakly RyR-positive. Calibration bar: 100 μm (A, B); 50 μm (a, b, c, d)

The ovarian distribution of InsP_3_Rs at the early infantile stage is depicted in Fig. 6A. For the most part, the ovarian InsP_3_Rs staining resembles Ca_V_1.3, Ca_V_2.1, and Ca_V_2.2 staining at the same stage (see Fig. 10E_1_). Despite the drastic morphological changes suffered by the ovary, the pattern of InsP_3_Rs staining resembles that of the neonatal stage: Oocytes and granulosa cells from primary to early antral follicles show moderate cytoplasmic labeling (Fig. 6A, and a, b: *blue* and *red asterisks,* respectively). Stroma and early theca cells surrounding ovarian follicles also express InsP_3_R weakly. Nonetheless, disseminated groups of perifollicular cells from secondary and early antral follicles show the strongest immunostaining (Fig. 6A, and a, b: *yellow arrows;* see Fig. 10E_1_).

#### RyR immunostaining

The staining pattern of ovarian RyRs at the early infantile stage (Fig. 6B) undergoes radical changes compared to the neonatal ovary (cf. Fig. 3B). The staining pattern also differs radically from that of InsP_3_Rs at the same stage: The whole ovary expresses RyRs: Oocytes, from primordial to early antral follicles, show punctate cytoplasmic staining (Fig. 6B, c, d: *blue asterisks*). Granulosa cells from the same follicles are labeled, particularly at the plasma membrane and in the cytoplasm closer to the oocyte (Fig. 6c, d: *blue arrows*). Immature theca cells, which begin to organize around secondary and early antral follicles, are moderately RyR-positive (Fig. 6c, d: *yellow arrows*. The fluorescence immunolabeling of Ca^2+^ channel subtypes in the ovary at postnatal day eight is summarized in the bar plots illustrated in Fig. 10 A_1_-F_1_.

### Expression of Ca^2+^ channels during the late infantile period (PND 16)

#### Ca_V_1.2 *(α1C)* immunostaining

**Fig. 7:**
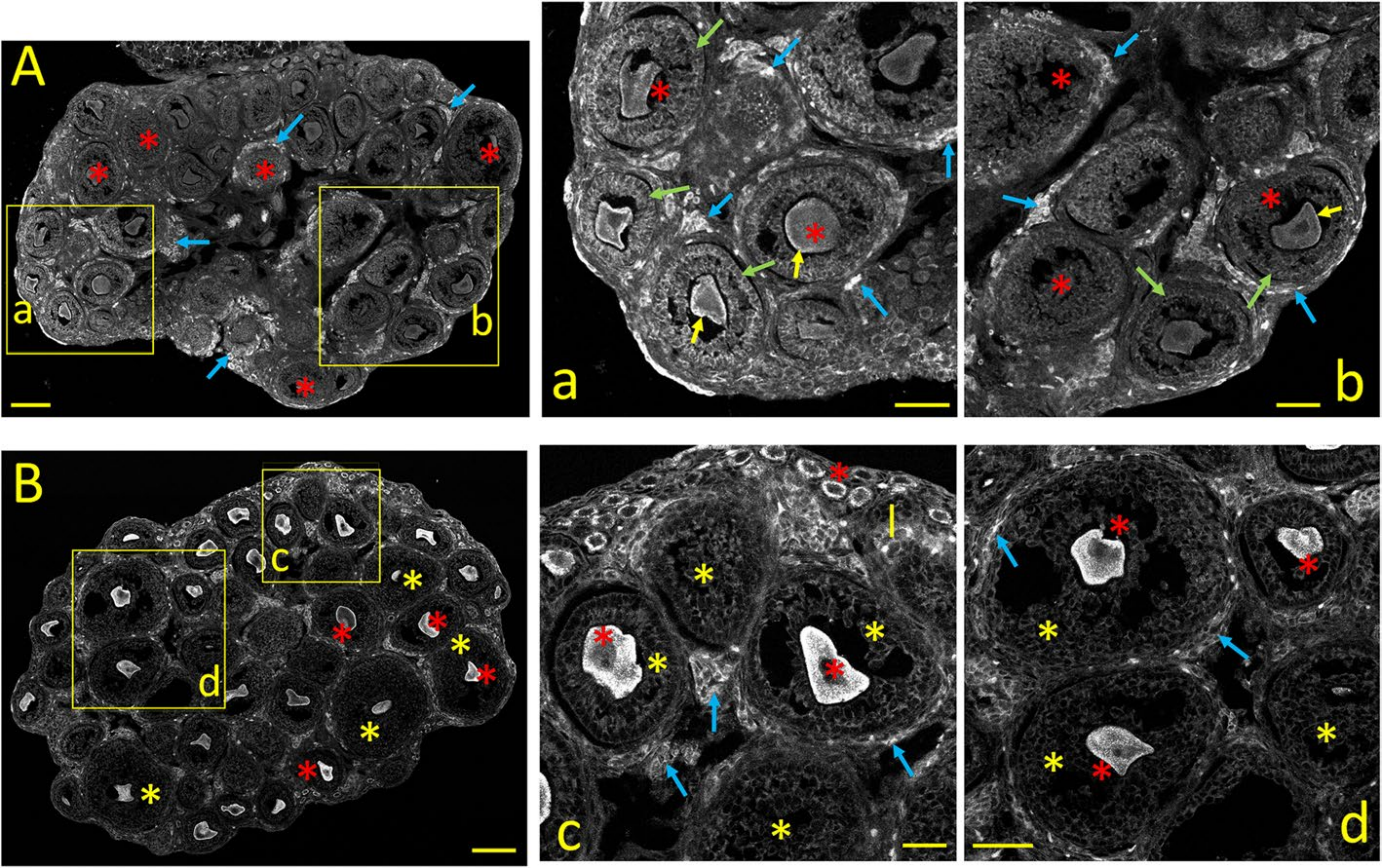
Tissue distribution of specific immunolabelling for Ca_V_1.2 and Ca_V_1.3 voltage-gated Ca^2+^ channels during the late infantile ovary (PND 16)**. A:** Ca_V_1.2 specific immunostaining. **a**,** b:** Enlarged images from the square areas indicated in **A**. *red asterisks*: oocytes and granulosa cells from primordial to early antral follicles are moderately stained. *yellow arrows:* Some oocytes show a strong signal near the plasma membrane. *green arrows:* granulosa cells, adjacent to the basal lamina are more intensely labeled. *blue arrows*: patched of perifollicular cells surrounding tertiary and antral follicles show the strongest Ca_V_1.2 staining. **B:** Ca_V_1.3 specific immunostaining. **c**,** d:** Enlarged images from the square areas indicated in B. *red asterisks:* oocytes, from primordial to pre-antral and antral follicles, show intense cytoplasmic immunostaining at this stage. *yellow asterisks:* granulosa cells from all follicle stages are Ca_V_1.3 negative. *blue arrows:* patches of perifollicular stromal cells show moderate Ca_V_1.3 expression. Calibration bar: 100 μm (A, B); 50 μm (a, b, c, d)

The expression pattern of Ca_V_1.2 (α1C) in the late infantile stage of the ovary (PND 15-20) is exemplified in Fig. 7A. Here, the ovary begins to show distinct signs of maturity: the *zona pellucida* is visible in some oocytes, and a distinct separation between GCs and theca cells becomes apparent. The hilus begins to form, and many secondary, tertiary, and antral follicles are visible (Fig. 7A and a, b: *red asterisks*). Groups of remaining primordial follicles are visible near the ovarian cortex. Nevertheless, antral follicles in the ovarian medulla indicate incomplete ovarian maturity. These follicles move close to the ovarian cortex during the more advanced peri-pubertal and mature stages. In general, the staining pattern of Ca_V_1.2 in the late infantile stage resembles that of the early infantile stage: Oocytes and granulosa cells from primordial to early antral follicles are moderately stained Fig. 7A and a, b: *red asterisks),* with some oocytes showing stronger signal near the periphery (Fig. 7a, b: *yellow arrows*). Interestingly, the first layer of granulosa cells, adjacent to the basal lamina, are more intensely labeled than the rest (Fig. 7a, b: *green arrows*). This pattern of Ca_V_1.2 immunostaining of granulosa cells was reported in the adult ovary [1]. Robust Ca_V_1.2 staining is also visible in incomplete layers of perifollicular cells surrounding tertiary and antral follicles (Fig. 7a, b: *blue arrows*; see Fig. 10A_2_). Unstained stromal cells occupy the hilum and the interfollicular area.

#### Ca_V_1.3 *(α1D)* immunostaining

The ovarian distribution of Ca_V_1.3 immunostaining at the late infantile stage is illustrated in Fig. 7B. Ca_V_1.3 staining is very different from that of Ca_V_1.2 (cf. Fig. 7A and B). The Ca_V_1.3 cytoplasmic staining of oocytes from primordial to pre-antral and antral follicles is particularly robust and patchy (Fig. 7B and c, d: *red asterisks*). In comparison, granulosa cells almost completely lack Ca_V_1.3 immunostaining (Fig. 7c, d: *yellow asterisks;* see Fig. 10B_2_). This result is intriguing because Ca_V_1.2 and Ca_V_1.3 s generate very similar L-type Ca^2+^ currents. Perifollicular stromal cells (presumably developing theca cells) partially surrounding pre-antral and antral follicles show mild Ca_V_1.3 expression (Fig. 7b and c, d *blue arrows;* Fig. 10B_2_).

#### Ca_V_2.1 *(α1A)* immunostaining

**Fig. 8:**
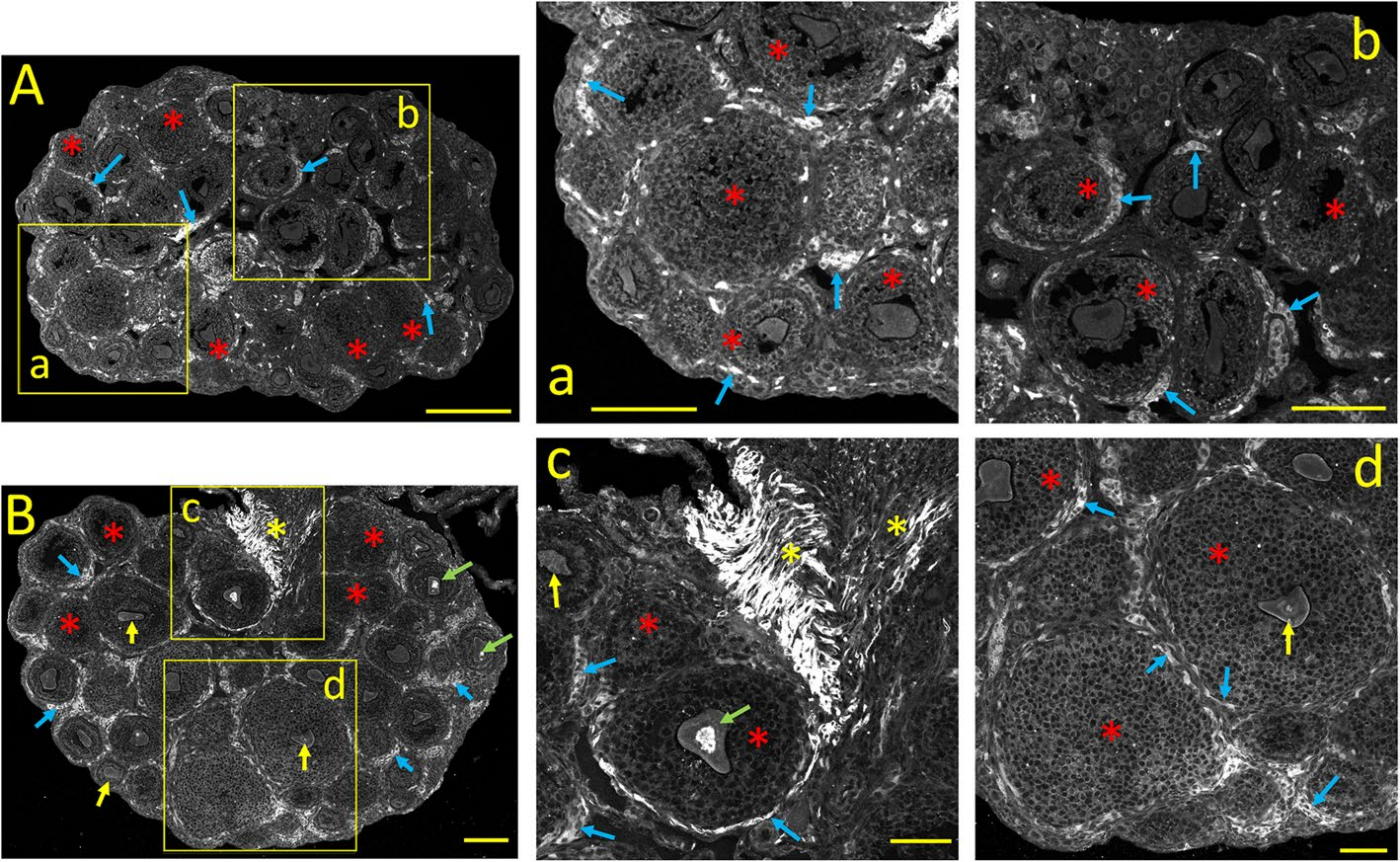
Tissue distribution of specific immunolabelling for Ca_V_2.1 and Ca_V_2.2 voltage-gated Ca^2+^ channels in the late infantile ovary (PND 16). **A:** Ca_V_2.1 specific immunostaining. **a**,** b:** Enlarged images from the square areas indicated in A. *red asterisks:* oocytes and granulosa cells from primary, secondary, pre-antral, and antral follicles are express Ca_V_2.1 weakly. *blue arrows:* in contrast, patches of perifollicular cells forming incomplete envelopes around secondary, early antral, and antral follicles are strongly stained. **B:** Ca_V_2.2 specific immunostaining. **c**,** d:** Enlarged images from the square areas indicated in B. Weak to moderate Ca_V_2.2 labeling is visible throughout the ovary. *red asterisks:* weakly stained granulosa cells. *yellow arrows:* most oocytes are weakly stained. *green arrows*: some oocytes display patches of intense labeling. *blue arrows:* patches of strongly Ca_V_2.2 positive perifollicular cells form an incomplete envelope around early antral and antral follicles. *yellow asterisks* strongly Ca_V_2.2 positive bundles of smooth muscle cells seen at the hilum. Calibration bar: 100 μm (A, B); 50 μm (a, b, c, d)

The staining pattern of Ca_V_2.1 during the late infantile stage is shown in Fig. 8A. Besides the noticeable differences resulting from ovarian growth and maturation, the main difference between the early and late infantile period is the comparatively weaker Ca_V_2.1 staining of oocytes and granulosa cells from primary, secondary, pre-antral, and antral follicles (see Fig. 8A and a, b: *red asterisks;* cf. Fig. 5A and Fig. 8A), while groups of fat cells forming incomplete envelopes of perifollicular cells around secondary, early antral, and antral follicles (possibly immature theca cells) are intensely stained (Fig. 8A and a, b: *blue arrows* see also Fig. 10C_2_).

#### Ca_V_2.2 *(α1B)* immunostaining

The immunostaining pattern of Ca_V_2.2 during the late infantile stage (Fig. 8B) differs markedly from that of other Ca_V_ channel subunits from the same developmental stage. This phase represents a clear step towards maturation from the pattern observed at the neonatal and early infantile stages: Ca_V_2.2 labeling is moderate throughout the ovary, including oocytes, stroma, theca, and granulosa cells from early antral and antral follicles (Fig. 8B and c, d). While most oocytes are weakly stained (Fig. 8B and c, d*; yellow arrows),* some display patches of intense labeling (Fig. 8B and c, *green arrows).* Nonetheless, plump stromal cells forming an imperfect envelope around early antral and antral follicles are strongly Ca_V_2.2 positive (Fig. 8B and c, d: *blue arrows*; see also Fig. 10D_2_). This staining pattern is reminiscent of the network of Ca_V_2.2-positive smooth muscle cells that surround mature follicles from the adult ovary [1]. The assumption that these cells are immature perifollicular smooth muscle cells is supported by the strong Ca_V_2.2 staining of bundles of smooth muscle cells at the hilum and other extra-ovarian structures (Fig. 8B and c: *yellow asterisks*).

#### InsP_3_R immunostaining

**Fig. 9:**
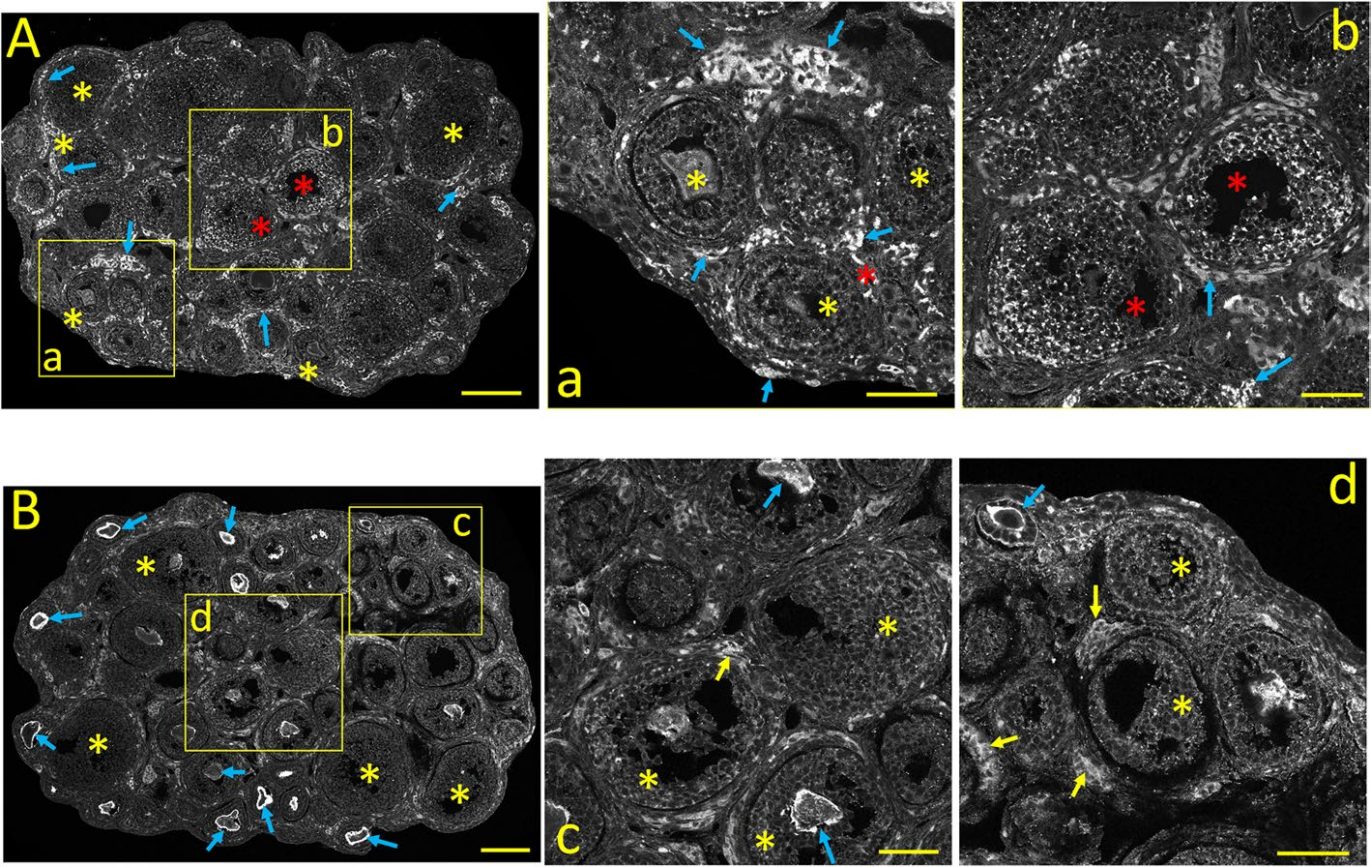
Tissue distribution of specific immunolabelling for Ca^2+^ release channels InsP_3_Rs and RyRs in the late infantile ovary (PND 16). **A:** InsP_3_R-specific immunostaining. **a**,** b:** Enlarged images from the square areas indicated in A. *yellow asterisks:* most oocytes and granulosa cells from primary to antral follicles are weakly stained. *red asterisks:* the granulosa cells of some follicles show intense “punctate” staining. *blue arrows:* strongly stained clusters of perifollicular cells surrounding secondary, early antral, and antral follicles. **B:** RyR-specific immunostaining. **c**,** d:** Enlarged images from the square areas indicated in B. *yellow asterisks*: granulosa cells are weakly stained, regardless of the follicle maturation. *blue arrows:* oocytes are strongly RyR-positive with aggregates of immunolabelling close to the plasma membrane. *yellow arrows:* clusters of fat perifollicular cells around early antral and antral follicles show moderate RyR staining. Calibration bar: 100 μm (A, B); 50 μm (a, b, c, d)

**Fig. 10:**
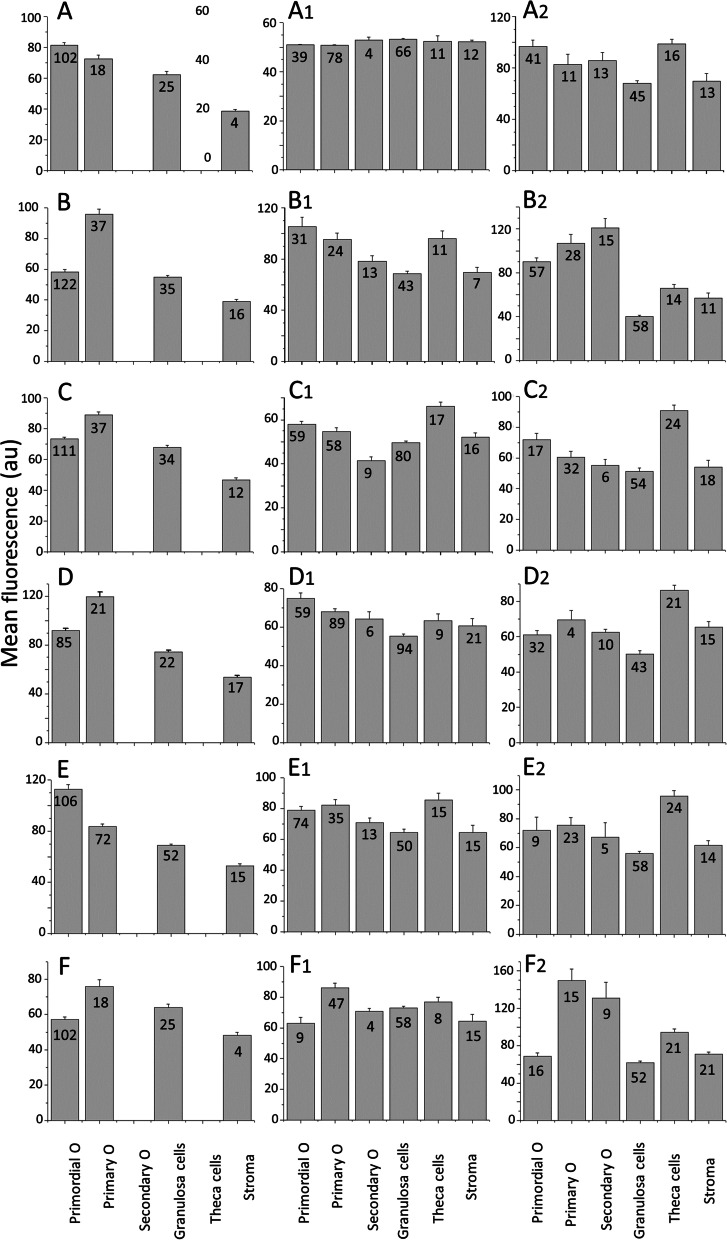
Quantification of specific immunolabelling of voltage-gated and intracellular release Ca^2+^ channels in the neonatal (left column), early infantile (middle column), and late infantile (right column) ovary. The fluorescence intensity of voltage-gated Ca_V_1.2 (**A**,** A**_**1**_,** A**_**2**_), Ca_V_1.3 (**B**,** B**_**1**_,** B**_**2**_), Ca_V_2.1 (**C**, **C**_**1**_,** C**_**2**_), and Ca_V_2.2 (**D, D**_**1**_,** D**_**2**_), as well as InsP_3_R (**E**,** E**_**1**_,** E**_**2**_) and RyR (**F**,** F**_**1**_,** F**_**2**_) from the different ovarian structures (primordial, primary, and secondary oocytes, granulosa cells, theca cells, and interfollicular stroma), was measured. The mean fluorescence intensities (arbitrary units) were plotted as bar graphs. **A-F**: PND3. **A**_**1**_**-F**_**1**_: PND8. **A**_**2**_**-F**_**2**_: PND16. The standard error of the mean of each bar is indicated and the number of objects measured of each type. The differences between bars are statistically significant (*p*<0.05), except for a few bars in Figures A1, D1, and F1

The distribution pattern of InsP_3_Rs in the late infantile ovary (Fig. 9A) shows substantial differences in InsP_3_Rs expression between the early and the late infantile ovary (cf. Figs. 6A and 9A). First, oocytes and granulosa cells from primary to antral follicles are comparatively less intensely stained (Fig. 9A and a, b: *yellow asterisks*). However, the granulosa cells of some early antral and antral follicles show intense punctate staining (Fig. 9b: *red asterisks*). This puncta of cytoplasmic InsP_3_R staining are likely clusters of the endoplasmic reticulum. InsP_3_R puncta can also be seen on scattered granulosa cells of other follicles. Finally, in the early and the late infantile stage, clusters of plump stromal cells surrounding secondary, early antral, and antral follicles are intensely stained (Fig. 9A and a, b: *blue arrows*; see also Fig. 10E_2_).

#### RyR immunostaining

The RyRs expression pattern during the late infantile stage (Fig. 9F) undergoes significant changes compared with the early infantile stage (Fig. 6A): granulosa cells are weakly stained, regardless of the stage of follicle maturation (Fig. 9B and c, d: *yellow asterisks*). Most oocytes from primary follicles are strongly RyR-positive with immunolabelling distinctly aggregated close to the plasma membrane (Fig. 9B and c, d: *blue arrows*). Clusters of perifollicular stromal cells (presumably immature theca or smooth muscle cells) around early antral and antral follicles are moderately stained (Fig. 9c, d: *yellow arrows*), while stromal cells and blood vessels are unstained. The mean fluorescence immunostaining of ovarian Ca^2+^ channel subtypes at postnatal day 16 are compared in the bar plots of Fig. 10A_2_ to 10F_2_.

## Discussion and Conclusions

The ovarian expression of voltage-gated plasma membrane Ca^2+^ channels and Ca^2+^-release channels (InsP_3_R and RyR) is discussed here in the context of the immature ovary's neuroendocrine landscape. The ovary is independent of pituitary gonadotropins during the neonatal stage (PND 0-7). Therefore, early follicular development relies on paracrine and autocrine signals. Some of these factors belong to the transforming growth factor-beta (TGF-β) superfamily [17, 25]: Bone Morphogenetic Protein 15 (BMP-15), Growth Differentiation Factor 9 (GDF-9), TFGβ-1, and Activins [29]. The oocyte secretes BMP-15 and GDF-9, allowing proliferation and differentiation of granulosa cells [32]. GDF-9 is essential for primordial follicle formation, recruitment to primary follicles, and progression to secondary follicles, while BMP-15 inhibits FSH action in granulosa cells, preventing further maturation of preovulatory follicles. Other local factors include Kit Ligand or Stem Cell Factor (KL, SCF), Basic Fibroblast Growth Factor–2 (FGF-2), Bone Morphogenetic Protein 4 (BMP-4), Leukemia Inhibitory Factor (LIF), and Keratinocyte Growth Factor–7 (FGF-7 [34];. The Anti-Müllerian hormone (AMH), secreted by granulosa cells from primary follicles, prevents excessive recruitment of primordial follicles [36]. Conversely, insulin recruits primordial follicles [36]. The transition from primary to secondary follicles also requires Nerve Growth Factor (NGF) provided by intra-ovarian extrinsic innervation [23].

Therefore, during the neonatal stage, folliculogenesis is controlled by protein growth factors secreted by the oocytes, immature granulosa cells, and nerve terminals, probably by exocytosis, mediated by Ca^2+-^ entry through voltage-gated Ca^2+^ channels (Ca_V_) and Ca^2+^ release from intracellular Ca^2+^ stores, by way of InsP_3_Rs and RyRs. Consequently, it is not surprising that oocytes from primordial and primary follicles express several types of Ca^2+^-mobilizing ion channels to support this task. Immature granulosa cells also express various types of Ca^2+^-selective channels. In contrast, stromal cells do not express Ca^2+^ channels at this stage.

Near the end of the neonatal stage, the pituitary begins to secrete FSH, and granulosa cells from primary follicles begin to express LH and FSH receptors. LHR transcripts are present from postnatal days 5, while FSHR transcripts are detectable from day 3, peaking by day 10. This early increase of LH- and FSH-receptor mRNAs in the immature mouse ovary is dependent on gonadotropin. Subsequent expression of LH-receptor is gonadotropin-dependent, while FSH-receptor mRNA continues increasing independently of gonadotropins [24].

FSH stimulates granulosa cell proliferation and differentiation. It also recruits primordial follicles and promotes their transition to secondary follicles [13, 23]. FSH binding to its receptor increases intracellular cAMP concentration, activating protein kinase A (PKA). Cross-talk between PKA and protein kinase C (PKC) can trigger Ca^2+^ influx and intracellular Ca^2+^ release [4, 11, 20]. It has been reported that activation of a subtype of FSH receptor, containing a growth factor type I receptor motif, elevates [Ca^2+^]_i_. This FSH-induced [Ca^2+^]_i_ rise is inhibited by removing external Ca^2+^ and after incubation with the Ca^2+^-channel blocker diltiazem, suggesting that FSH can increase [Ca^2+^]_i_ directly through the opening of L-type Ca^2+^ channels [35]. Our results show that immature granulosa cells not only express the Ca_V_1.2 and Ca_V_1.3 subunits that underlie L-type Ca^2+^-channels but other Ca^2+^-selective channels capable of supporting robust Ca^2+^ signaling. Furthermore, during the neonatal period, adrenergic nerve fibers arrive in the ovary [23]. By releasing catecholamines and vasoactive intestinal polypeptide (VIP), which bind to β2-adrenergic and VIP receptors on immature granulosa and theca cells, these nerve fibers stimulate steroidogenesis [23]. Ca_V_1.2 and Ca_V_2.1 Ca^2+^ channels, expressed in ovarian nerve fibers and autonomic neurons, could regulate the release of these neurotransmitters.

Late in the neonatal stage, the plasma concentration of gonadotropins increases further, reaching peak levels by the early infantile period (PND 12 [8];). As it turns out, gonadotropin secretion is stimulated by the luteinizing hormone-releasing hormone (LHRH) secreted by peptidergic neurons in the hypothalamus. During the second postnatal week, gamma-aminobutyric acid (GABA), the primary inhibitory neurotransmitter in the CNS, elicits *excitatory* postsynaptic potentials in these neurons, thus increasing their firing rate and promoting LHRH release. The reverse polarity of synaptic potential is due to the relatively high intracellular chloride concentration of LHRH neurons. Their hyperactivity is also explained by the absence of circulating ovarian estrogens (E2), which inhibit these hypothalamic neurons. Plasma E2 levels are low because the aromatase activity of immature granulosa cells is insufficient to convert androgens into estrogens, and alpha-fetoprotein (AFP) in the bloodstream sequesters any small amount of E2 produced [26]. The enhanced secretion of LHRH during the late neonatal stage promotes the release of pituitary gonadotropins.

During the early infantile stage (PND 8-14). Fewer primordial follicles remain near the cortex, while secondary follicles grow underneath. Subsequently, by PND 10, early antral follicles, characterized by a small antrum, begin to appear [10]. However, these early antral follicles are immature compared to those of the cycling adult (see above). Progression from secondary to antral follicles marks the ovary's transition from pituitary-independent in the neonatal period to pituitary-dependent in the early infantile period. As the expression of LH and FSH receptors in theca and granulosa cells increases gradually throughout the early infantile period [24], LH/FSH serum levels also increase, reaching peak levels by PND 12-14 [23]. The combination of increasing LH/FSH levels and increased expression of LH/FSH receptors [24] supports the transformation of secondary follicles into early antral follicles [13, 14].

Our immunostaining data suggest that the transition from neonatal to early Infantile stage coincides with a diminished expression of Ca_V_, InsP_3_R, and RyR in the oocytes (except for those from the remaining primordial follicles) and an increased expression in the granulosa cells and stromal cells surrounding secondary follicles (possibly immature theca cells). This developmental shift of Ca^2+^ channel expression from oocytes to granulosa and perifollicular cells likely reflects the declining role of oocytes in controlling follicular development and the more significant role of granulosa cells as they become increasingly responsive to pituitary gonadotropins.

Further changes occur in the ovary during the late infantile period (PND 15-20): GABAergic neurotransmission to LHRHergic hypothalamic neurons switches from excitatory to inhibitory as intracellular chloride concentration in these neurons diminishes. This inhibitory drive reduces LHRH hypothalamic secretion. LHRH release also decreases because, starting at PND 12*,* granulosa cells convert more efficiently the androgens produced by theca cells into E2, and AFP vanishes from circulation. Therefore, E2 becomes available to exert negative feedback on LHRHergic neurons [23]. These elements combine to explain the sudden drop in LH/FSH release [6].

Does the failing pituitary influence affect the ovarian expression pattern of Ca_V_, InsP_3_R, and RyR during the late infantile stage? In general, the changes in the expression pattern of Ca^2+^ channels in the ovary continue that observed at 8 PND: a reduced expression in oocytes from pre-antral and antral follicles and an increased expression in granulosa and theca cells. An exception is the expression of Ca_V_1.3 Ca^2+^ channels: After a diminished oocyte immunoreactivity during the early infantile period, relative to other ovarian cells, Cav1.3 expression increases in the late infantile stage, becoming the dominant staining, while the Ca_V_1.3 expression of granulosa cells almost disappears (Fig. 7B, see also Fig. 10B_2_).

The changes in the expression pattern of Ca^2+^-selective channels during the *late infantile stage* suggest that oocytes are no longer the main ones responsible for generating Ca^2+^ signals at this stage because theca and granulosa cells control the growth and maturation of follicles. Granulosa cells adjacent to the basal lamina express more Ca_V_1.2 than the rest, suggesting a role of Ca^2+^ entry in converting androgens into estrogen. Ca_V_2.2-positive cells surrounding early antral and antral follicles are likely immature smooth muscle cells. These cells completely enclose ovarian follicles in the adult, playing an essential role in ovulation [18]. The release of local factors regains relevance during the *late infantile period*. At PND 15, granulosa cells begin to release activin and inhibin [28, 39], two members of the TGFB superfamily that promote and inhibit, respectively, pituitary synthesis and FSH secretion. As inhibin production increases gradually, LH/FSH levels decrease [9, 15]. Conversely, activin promotes aromatase activity, antral cavity formation, FSH receptor expression, and granulosa cell proliferation [7, 39].

Transient elevations of [Ca^2+^]_i_ occur in several cell types before and during mitosis [30]. For example, In proliferating neuroendocrine cells, Ca^2+^ fluxes underlying these [Ca^2+^]_i_ signals are mediated by Ca_V_1.2 and Ca_V_1.3 Ca^2+^ channels [19]. The proliferation of granulosa cells at the infantile stage likely involves the participation of voltage-gated Ca^2+^ channels and InsP_3_Rs. Follicle maturation continues during the late infantile stage, despite the declining FSH levels, because activin promotes LH/FSH receptor expression in granulosa cells, significantly increasing LH/FSH sensitivity [12]. During this stage, prolactin and growth hormone (GH) secretion in the pituitary facilitates LH/FSH actions in the ovary.

At more advanced stages, during the juvenile period (PND 21-32)**,** FSH secretion remains low because GABAergic inhibitory transmission on LHRH neurons persists [22, 25]. The ovary produces inhibin and E2, negative feedback regulators of pituitary gonadotropin secretion. The minimum serum levels of FSH are reached by PND 30, which correlates with the peak of follicle atresia. Nonetheless, other follicles continue their development despite the low levels of serum FSH, probably because FSH/LH receptor density reaches its highest levels in granulosa cells, resulting in an exquisite sensitivity to FSH/LH.

One could speculate that Ca^2+^ signaling would be robust in cells with greater expression of Ca^2+^ channels: i. e. oocytes during the neonatal stage and theca and granulosa cells during the infantile stage. This assumption can be tested using Ca^2+^ imaging in living ovarian slices [1]. We plan to compare the expression pattern of Ca^2+^ channels throughout the juvenile, peri-pubertal (PND 33-37), and pubertal stages (PND 38-46). Of particular interest is the peri-pubertal period, characterized by daily surges of LH that facilitate antral follicles' maturation into ovulatory follicles. As oocytes become competent for ovulation and fertilization at puberty, they must recuperate their Ca^2+^ signaling capabilities since vigorous and sustained [Ca^2+^]_i_ signals are essential during fertilization and have critical roles during early events of egg activation and egg-to-embryo transition [3]. The increased oocyte expression of Cav1.3 and RyRs in early antral and antral follicles during the late infantile period could be the prelude of this essential functional adaptation.

## Conclusions

Oocytes and granulosa cells in the neonatal ovary express an assortment of Ca^2+^ selective channels capable of supporting Ca^2+^- dependent exocytosis of autocrine and paracrine growth factors. In contrast, the early infantile period coincides with a diminished expression of Ca^2+^- permeable channels in the oocyte and increased expression in the granulosa and immature theca cells, which control folliculogenesis in response to gonadotropins.

## Methods

### Animals

CD1 mice from postnatal days (PND) 3, 8, and 16 were employed. Three mice of each age were used. They were maintained under controlled conditions of light and temperature (12 h light, 12 h dark) and free access to food and water. Mice were maintained in the animal facility and fed ad libitum. All animals were sacrificed at noon. For the morphologic descriptions of follicles and the immature ovary, we used the definitions from Picut [25].

#### Immunofluorescence of frozen ovarian sections

Mice of the same age were injected with a terminal dose of pentobarbital (60 mg/kg, IP), perfused transcardially with phosphate-buffered saline (PBS, 0.1M), and then ice-cold 4% paraformaldehyde in PBS. Ovaries were removed, cleaned, and post-fixed in 4% paraformaldehyde in PBS for 3 hrs at 4^o^ C and then placed in PBS with 10%, 20%, and 30% sucrose (overnight incubation with each) to prevent freeze damage through (water- ice crystal formation. Finally, they were included in Tissue-Tek (Sakura Finetek) for sectioning (Leica Microsystems cryostat CM1900, Wetzlar, Germany). Frozen sections (10 *μ*m in thickness) were mounted on *Superfros*t glass slides (Fisher Scientific), washed with PBS and incubated with Saline-Sodium Citrate 2x solution at 65°C for 45 min, and placed in PBS blocking solution (with 3% donkey normal serum) with 0.1% triton 100 for 60 min at room temperature for permeabilization. Three sections from each ovary were mounted and immunostained from at least two different animals. Then, sections were incubated for 24 hrs. in a humid chamber at 4°C with one of the primary rabbit antibodies from Alomone Labs (Jerusalem, Israel): anti-Ca_V_ 2.1 (*α*1A; validation number 2039764, lot # AN-09), anti-Ca_V_ 2.2 (*α*1B; validation number 2039766, lot # AN-015), anti-Ca_V_ 1.2 (*α*1C; validation number 2039771, lot # AN-19) or anti-Ca_V_ 1.3 (*α*1D; validation number 2039775, lot # AN-09**:** dilution 1:100), rabbit anti-InsP_3_R (I, II, III) H-300 Santa Cruz Biotechnologies (sc-28613; dilution 1:50) and mouse anti-RyR C3-33, (ab2827; dilution 1:20) from ABCAM. Frozen sections were washed and incubated at room temperature for 2 hrs with F (ab')2 Alexa 488 fraction of donkey anti-rabbit IgG (1:500 dilution, validation number 2340586 cat # 705–006-147; Jackson ImmunoResearch Lab, West Grove, PA) or with the F (ab')2 Alexa 647 fraction of donkey anti-mouse IgG (H + L) in PBS (validation number 2340386 cat # 705–006-152; Jackson ImmunoResearch Lab; dilution 1:500) Finally; the sections were washed and mounted with Dako Glycergel (Dako North American, Inc., CA). Several controls were required for reliable immunolabeling: Anti-Ca_V_ primary antibodies were pre-adsorbed with the corresponding synthetic antigenic peptide (2 *μ*g peptide per 1 *μ*g antibody). This procedure completely blocked specific immunofluorescence. Also, incubation with the secondary antibody alone gave a weak, nonspecific fluorescence (see Supplementary Figure 1).

Ovarian sections were viewed by confocal microscopy. Two representative sections from each age and different mice were chosen for display and quantitative analysis. Images were acquired with an LSM 800 inverted microscope (Zeiss, Jena, Germany) with a 40X Plan APO objective (oil immersion 1.3 NA; Zeiss). Samples were excited with either a 488 nm or 647 nm laser and acquired with GaAsP detectors. For the reconstruction of each ovarian section, between 70 and 120 images (512 × 512 pixels) were acquired using the "tile scan mode" and a programmable motorized stage. The confocal microscope settings (laser power 1%, pinhole airy unit =1, main gain 600 V) were the same for all images. Raw image intensity was normalized so that no more than 0.01% of pixels were saturated. Some figures were digitally processed (i.e., multiplied by a factor of 1.5) to emphasize certain aspects or weakly stained structures. As a result, small areas in some images may appear saturated. The fluorescence intensity of the different ovarian structures was measured in two slices from each age and antibody (36 slices in total), using *Fiji is Just* software (Image J 1.53c). For this purpose, areas of interest were drawn by hand to analyze the mean fluorescence of all primordial, primary, and secondary oocytes, granulosa cells, theca cells, and interfollicular stroma separately. Primordial follicles have a single layer of flattened pre-granulosa cells which are difficult to measure; therefore, granulosa cells belong to primary, secondary, and preantral follicles. In Fig. 10, the mean fluorescence values ± standard error were plotted as bar graphs for comparison. The Scheffé posthoc test was used, allowing multiple comparisons of group means.

## Supplementary Information

**Additional file 1: Supplementary Figure 1.** Comparison of nonspecific and specific fluorescence. **A:** Ca_V_1.2 *(α1C)* immunostaining of a frozen ovarian section at PND 16. After 24 hrs incubation with the anti-Ca_V_1.2 primary rabbit antibody, the section was incubated for 2 hrs with the Alexa 488 donkey anti-rabbit IgG. **B:** Another ovarian section from the same stage was incubated for 2 hrs with the donkey anti-rabbit IgG alone to characterize nonspecific staining. **C:** RyR immunostaining of a frozen ovarian section at PND 3. After 24 hr incubation with the anti-RyR primary mouse antibody, the section was incubated with the Alexa 647 donkey anti-mouse IgG. **D:** Another ovarian section from the same stage was incubated for 2 hrs with the secondary donkey anti-mouse IgG alone to demonstrate nonspecific staining. Confocal microscope settings (laser power, master gain, pinhole size) were the same for all images, and pixel values of raw images were multiplied by the same factor (1.5). Calibration bars: 100 μm.
