# Supplementary figures and images for: Expression of voltage-gated Ca2+ channels, Insp3Rs, and RyRs in the immature mouse ovary
Source: J Ovarian Res. 2022 Jul 22;15:85. doi: 10.1186/s13048-022-01015-y (PMC9306205; doi:10.1186/s13048-022-01015-y)

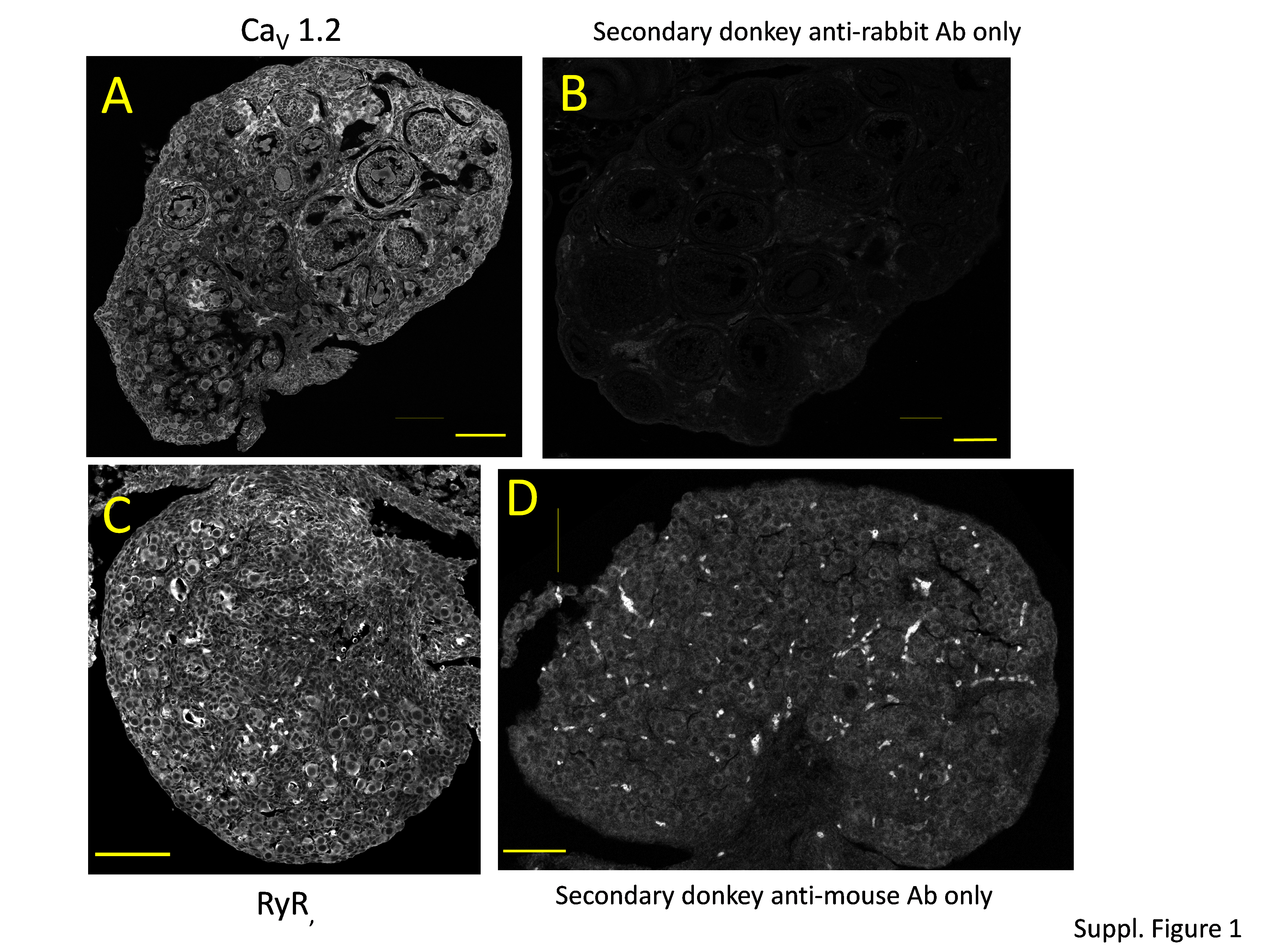

Supplement: Supplementary file 1 — Additional file 1: Supplementary Figure 1. Comparison of nonspecific and specific fluorescence. A: CaV1.2 (α1C) immunostaining of a frozen ovarian section at PND 16. After 24 hrs incubation with the anti-CaV1.2 primary rabbit antibody, the section was incubated for 2 hrs with the Alexa 488 donkey anti-rabbit IgG. B: Another ovarian section from the same stage was incubated for 2 hrs with the donkey anti-rabbit IgG alone to characterize nonspecific staining. C: RyR immunostaining of a frozen ovarian section at PND 3. After 24 hr incubation with the anti-RyR primary mouse antibody, the section was incubated with the Alexa 647 donkey anti-mouse IgG. D: Another ovarian section from the same stage was incubated for 2 hrs with the secondary donkey anti-mouse IgG alone to demonstrate nonspecific staining. Confocal microscope settings (laser power, master gain, pinhole size) were the same for all images, and pixel values of raw images were multiplied by the same factor (1.5). Calibration bars: 100 μm. [file 13048_2022_1015_MOESM1_ESM.png]
